# Kinetics and products of *Thermotoga maritima* β-glucosidase with lactose and cellobiose

**DOI:** 10.1007/s00253-024-13183-6

**Published:** 2024-05-29

**Authors:** Geert A. ten Kate, Peter Sanders, Lubbert Dijkhuizen, Sander S. van Leeuwen

**Affiliations:** 1 Microbial Physiology, Groningen Biomolecular Sciences and Biotechnology Institute (GBB), University of Groningen, Nijenborgh 7, 9747 AG Groningen, The Netherlands; 2 Eurofins Expertise Centre for Complex Carbohydrates and Chemistry, PO Box 766, 8440 AT Heerenveen, The Netherlands; 3 Department of Laboratory Medicine, University of Groningen, University Medical Center Groningen, Hanzeplein 1, EA30, 9713 GZ Groningen, The Netherlands; 4 Present Address: Royal FrieslandCampina, Stationsplein 4, 3818 LE Amersfoort, The Netherlands; 5 Present Address: CarbExplore Research BV, Zernikelaan 8, 9747 AA Groningen, The Netherlands; 6 Present Address: Van Hall Larenstein, University of Applied Sciences, Agora 1, P.O. box 1528, 8901 BV Leeuwarden, The Netherlands

**Keywords:** Galacto-oligosaccharides, Gluco-oligosaccharides, Thermostable, Glucosidase, Galactosidase

## Abstract

**Abstract:**

Galacto-oligosaccharides (GOS) are prebiotic compounds that are mainly used in infant formula to mimic bifidogenic effects of mother’s milk. They are synthesized by β-galactosidase enzymes in a *trans*-glycosylation reaction with lactose. Many β-galactosidase enzymes from different sources have been studied, resulting in varying GOS product compositions and yields. The in vivo role of these enzymes is in lactose hydrolysis. Therefore, the best GOS yields were achieved at high lactose concentrations up to 60%*wt*, which require a relatively high temperature to dissolve. Some thermostable β-glucosidase enzymes from thermophilic bacteria are also capable of using lactose or *para* nitrophenyl-galactose as a substrate. Here, we describe the use of the β-glucosidase BglA from *Thermotoga maritima* for synthesis of oligosaccharides derived from lactose and cellobiose and their detailed structural characterization. Also, the BglA enzyme kinetics and yields were determined, showing highest productivity at higher lactose and cellobiose concentrations. The BglA *trans*-glycosylation/hydrolysis ratio was higher with 57%*wt* lactose than with a nearly saturated cellobiose (20%*wt*) solution. The yield of GOS was very high, reaching 72.1%*wt* GOS from lactose. Structural elucidation of the products showed mainly *β*(1 → 3) and *β*(1 → 6) elongating activity, but also some *β*(1 → 4) elongation was observed. The β-glucosidase BglA from *T. maritima* was shown to be a very versatile enzyme, producing high yields of oligosaccharides, particularly GOS from lactose.

**Key points:**

• *β-Glucosidase of Thermotoga maritima synthesizes GOS from lactose at very high yield*.

• *Thermotoga maritima β-glucosidase has high activity and high thermostability*.

• *Thermotoga maritima β-glucosidase GOS contains mainly (β1-3) and (β1-6) linkages*.

**Graphical Abstract:**

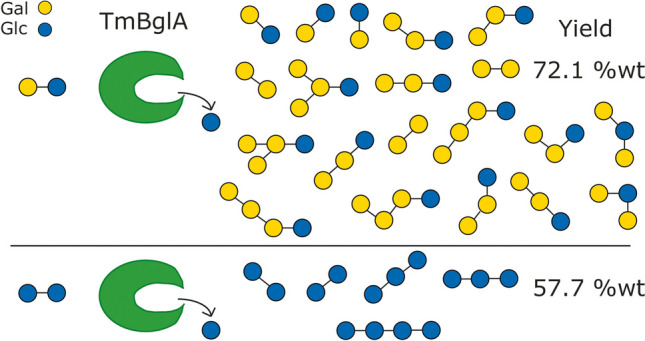

**Supplementary Information:**

The online version contains supplementary material available at 10.1007/s00253-024-13183-6.

## Introduction

Galacto-oligosaccharides (GOS) are synthesized from lactose via a *trans*-glycosylation reaction catalyzed by normally hydrolytic β-galactosidase enzymes (e.g., the *Bacillus circulans* β-galactosidase BgaD). These β-galactosidase enzymes belong to the glycoside hydrolase (GH) A class of glycoside hydrolase enzymes (www.CAZy.org), containing a TIM (β/α)_8_ barrel catalytic domain (Drula et al. 2022). Within this class, there are several families, based on amino acid sequence similarity. GH-A clan β-galactosidases (EC 3.2.1.23) belong to families GH1, 2, 35, 42, 50, 59, and 147. Various studies have identified a wide variety of structures in commercial GOS products (van Leeuwen et al. 2016), based on effective GOS-synthesizing enzymes (Nguyen et al. 2006; Cardelle-Cobas et al. 2008; Goulas et al. 2009; Rodriguez-Colinas et al. 2011; Yin et al. 2017a).

Not all compounds that are currently considered to have prebiotic properties pass through the mammalian digestive system entirely intact (Bindels et al. 2015; Yan et al. 2018). Using rat intestinal extracts, a commercial GOS mixture was hydrolyzed up to 50% (Ferreira-Lazarte et al. 2017). Particularly, GOS with a degree of polymerization (DP) of 2 and 3 were susceptible to hydrolysis. Still, addition of GOS to infant nutrition has been shown to shift the gut microbiome towards a higher amount of bifidobacteria in vivo (Oozeer et al. 2013). Also, in studies with adults and livestock, GOS bifidogenic activity has been observed (Jung et al. 2008; Davis et al. 2010; Vulevic et al. 2015; Alizadeh et al. 2016). Previous work has suggested that β-galactosidases from bifidobacteria have a preference for hydrolyzing *β*(1 → 6) and *β*(1 → 3) linkages (Cardelle-Cobas et al. 2011; Arreola et al. 2014), resulting in release of glucose and galactose as carbon sources for growth, rendering such GOS the most suitable for use in infant nutrition.

Most β-galactosidase enzymes that have a preference for the synthesis of *β*(1 → 3) or *β*(1 → 6)-linked GOS from lactose show only moderate yields (27–44%_wt_) (Arreola et al. 2014; Kittibunchakul et al. 2020; Füreder et al. 2020). The most commonly used commercial enzyme, *B. circulans* BgaD enzyme, reaches yields up to 63.3%_wt_ (Benjamins et al. 2014). The BgaD enzyme, however, produces mainly *β*(1 → 4)-linkages (Van Leeuwen et al. 2014). Therefore, we are evaluating other atypical β-galactosidase enzymes for the synthesis of novel GOS mixtures, reaching high GOS yields with predominantly *β*(1 → 3) and *β*(1 → 6) linkages.

Glucose oligosaccharides, containing *β*-linked residues, are less well studied, but early studies have shown promising properties (Rycroft et al. 2001; Kothari and Goyal 2015; Sahasrabudhe et al. 2016; Lee et al. 2020; Yu et al. 2023). Cello-oligosaccharides containing *β*(1 → 4)-linked glucose units are resistant to digestion, but are also not used by the gut microbiota (Yu et al. 2023). In a preliminary study, gentio-oligosaccharides, consisting of *β*(1 → 6)-linked glucose units, were shown to have prebiotic properties (Rycroft et al. 2001; Kothari and Goyal 2015). β-Gluco-oligosaccharides with *β*(1 → 3)-linked residues showed potential as prebiotic, but also stimulated the immune system (Sahasrabudhe et al. 2016; Yu et al. 2023). Most of these oligosaccharides are currently produced by partial acid hydrolysis or enzymatic hydrolysis of natural polysaccharides, like curdlan and laminarin (Sahasrabudhe et al. 2016; Lee et al. 2020; Yu et al. 2023).

A few studies have shown that β-glucosidase enzymes from extremophilic bacterial species are also capable of hydrolyzing lactose (Gabelsberger et al. 1993), even performing *trans*-glycosylation reactions (Goyal et al. 2001). Recently, the GOS product synthesized from lactose by the β-glucosidase from *Thermotoga naphthophila* was suggested to contain only one structure, i.e., 3′-galactosyllactose (Yang et al. 2018). Here, we report the analysis of the *trans*-glycosylation activity of the *T. maritima*–derived GH2 β-glucosidase (*r*TmBglA), using lactose and cellobiose as single substrates (both as donor and acceptor). *r*TmBglA displayed a highly effective *trans*-glycosylation activity with lactose (GOS; yield 411 g/L; 72.1%*wt*) and cellobiose (GlcOS; yield 115 g/L; 57.7%*wt*). Structural characterization of the *trans*-glycosylase product mixtures showed that with lactose and cellobiose, the *r*TmBglA enzyme had a preference for formation of *β*(1 → 3) and *β*(1 → 6) linkages. Lower levels of *β*(1 → 4) elongated products were also observed with both substrates, while higher relative *β*(1 → 4) activity was observed in the glucosyltransferase reaction with cellobiose than in the galactosyltransferase reaction with lactose.

## Materials and methods

### Materials

The *T. maritima* β-glucosidase A (TmBglA) was purchased from Megazyme Int (prod. nr. E-BGOSTM).

Expression vector pET100/D-TOPO-*r*TmBglA, containing the synthetic, recombinant *r*TmBglA encoding gene (446 aa sequence from UniProtBK accession number Q08638, BglA gene from *T. maritima* strain ATCC 43589/DSM 3109/JCM 10099/NBRC 100826/MSB, with added His6-tag; Supporting information contains full nucleotide sequence), was purchased from GeneArt (ThermoScientific) and transformed into Ca^2+^ competent *E. coli* BL21 star (DE3) cells by heat-shock treatment.

### Enzyme expression

*E. coli* BL21 star (DE3) containing the pET100/D-TOPO-*r*TmGblA plasmid was grown in 100-mL LB medium, containing 100 μg/mL ampicillin to an optical density (600 nm) of 0.75. The expression of the *r*TmBglA encoding gene was induced by isopropyl-β-d-thiogalactopyranoside (IPTG; 0.1 mg/mL final concentration), and cells were cultivated overnight at 17 °C. Cells were harvested by centrifugation (10,000 × g, 20 min) and subjected to chemical lysis using B-PER (Thermo Scientific, Pierce), containing lysozyme. Cell debris was centrifuged (15,000 × g, 20 min), and cell-free extract was subjected to heat treatment (75 °C, 10 min) to precipitate thermo-labile *E. coli* proteins, followed by centrifugation (10,000 × g, 20 min). The clear supernatant was used to further purify *r*TmGblA by Ni^2+^ affinity chromatography on Ni–NTA (Sigma-Aldrich). Protein was bound to the Ni–NTA column during incubation for 4 h at 4 °C. After washing the column with 25 mM Tris–HCl (pH 8.0) containing 1 mM CaCl_2_, protein was eluted with the same buffer containing 200 mM imidazole. The enzyme was desalted and concentrated with a 30 kDa cut-off centrifugal filter and reconstituted to 0.5-mL final volume in Milli-Q water. Protein concentration was determined in six repeats by Nanodrop 2000 spectrophotometer (Isogen Life Science) analysis in relation to a BSA calibration curve, using Milli-Q water as blank. Purity was verified by SDS-PAGE analysis.

### Enzyme reactions

Enzyme kinetics towards lactose and cellobiose was analyzed with the glucose oxidase/peroxidase assay (GOPOD), measuring glucose release to determine total units of activity (U). For lactose, 1 U of activity was defined as 1 μmol Glc released per min by 1 mg of enzyme. In case of cellobiose, one cycle of activity will release 2 Glc units; thus, 1 U is equal to the release of 2 μmol Glc per min by 1 mg of enzyme. Kinetic properties with lactose were analyzed in triplicate (*n* = 3), using a concentration range of 0.1–75 mM lactose incubated with 3.38 µg of *r*TmBglA enzyme in 150-µL incubations. Kinetics with cellobiose was analyzed with two triplicates (*n* = 6), using a concentration range of 1–250 mM cellobiose incubated with 3.38 µg of *r*TmBglA enzyme in 150-µL incubations.

Initial optimization experiments for GOS synthesis were performed with TmBglA obtained from Megazyme, using varying lactose concentrations (45 and 75%*wt*), pH (5 and 8.5), temperatures (60 or 90 °C), and enzyme units (2.5 or 5 U/g lactose). As a reference, incubation according to typical industrial production (57%*wt* lactose, 75 °C, pH 6.8, using 3.75 U/g lactose) was also taken along. It should be noted that lactose slurries were prepared by weighing 450 mg lactose and adding 550 μL buffer, 570 mg lactose, adding 430 μL buffer or 750 mg lactose, adding 250 μL buffer and heating to 90 °C for 10 min allowing slurry formation, followed by cooling to the desired reaction temperature. Subsequent optimization was performed with the purified synthetic *r*TmBglA gene product, using 3.75 U/g lactose and 57%*wt* lactose and varying pH (from 3 to 9) and temperatures (from 60 to 90 °C), to determine optimum pH and temperature values. Yield optimization experiments were performed using the previously determined reaction optima, with lactose concentrations varying from 50 to 65%*wt* lactose, prepared as described above*.* Final GOS synthesis at optimal yield was performed using 3.75 U/g lactose, with 57%*wt* lactose at 75 °C in a 25 mM NaOAc buffer (pH 5.5). Incubations with 20%*wt* cellobiose were performed at the same temperature and pH conditions, using the same amount of enzyme (3.75 β-galactosidase U/g substrate). Activity and GOS yields were monitored by profiling of galactose (Gal), glucose (Glc), and lactose against 5-point standard curves (80–1000 μM) using high performance anion exchange chromatography (HPAEC) coupled with a pulsed amperometric detector (PAD). GOS yields were determined by the difference between initial lactose and remaining lactose, corrected for hydrolysis derived from Gal formation.

### NMR spectroscopy

Product samples were lyophilized and exchanged twice with 99.9 atom% D_2_O (Cambridge Isotope Laboratories, Inc.). Finally, samples were dissolved in 650 μL D_2_O, containing internal acetone (δ^1^H 2.225). One-dimensional ^1^H NMR spectra were recorded on a Varian Inova 600 MHz spectrometer (NMR Department, University of Groningen) at a probe temperature of 25 °C. Spectra were recorded in 16–32 transients of 16 k data points and a sweep width of 4800 Hz. After zero filling to 32 k and Fourier transform, the spectra were phased manually, and a Whittaker Smoother baseline correction was applied in MestReNova 10.0.02 (MestRelabs, Santiago de Compostella, Spain).

### HPAEC-PAD

GOS and GlcOS incubations are diluted to 0.5 mg/mL initial substrate. Injections of 10-μL diluted GOS and GlcOS products, as well as five quantitative calibration standards, containing Gal, Glc, cellobiose, and lactose in a concentration range of 75 μM up to 1 mM, were profiled on a Dionex ICS-3000 workstation (Dionex, Amsterdam, The Netherlands), equipped with a CarboPac PA-1 column (250 × 2 mm, Dionex) and an ICS-3000 ED pulsed amperometric detector (PAD), using a complex gradient of A: 100 mM NaOH, B: 600 mM NaOAc in 100 mM NaOH, C: Milli-Q water, and D: 50 mM NaOAc. The fractionations were performed at 0.25 mL/min with 10% A, 85% C, and 5% D in 25 min to 40% A, 10% C, and 50% D, followed by a 35-min gradient to 75% A, 25% B, directly followed by 5-min washing with 100% B and reconditioning for 7 min with 10% A, 85% B, and 5% D.

## Results

### Enzyme purity assessment

The Megazyme enzyme was analyzed by SDS-PAGE to check purity (Supplementary Fig. S1A). Besides a strong band at the expected MW of ~ 52 kDa, bands of smaller proteins were observed in the preparation. An expression-vector plasmid containing a synthetic gene (GeneArt; Thermo Scientific) encoding *r*TmBglA (Q08638 with His6-tag) was purchased and successfully transferred into an expression host *E. coli* BL21 (DE3*) and expressed by IPTG induction. Purification with Ni–NTA affinity chromatography did not yield completely pure protein (Supplementary Fig. S1B); therefore, a heat-treatment step (75 °C, 10 min) followed by centrifugation to precipitate denatured protein prior to the Ni–NTA step was added, showing only one protein band on SDS-PAGE (Supplementary Fig. S1C).

The purified recombinant enzyme concentration was determined by UV280 nm absorption on a Nanodrop machine, relative to a BSA standard curve. From 100-mL *E. coli* culture expression, 3.38-mg active, pure *r*TmBglA enzyme was obtained. The purified *r*TmBglA protein was used for kinetic analysis with lactose and cellobiose as substrates and for GOS and GlcOS synthesis.

### Optimization experiments

**Fig. 1:**
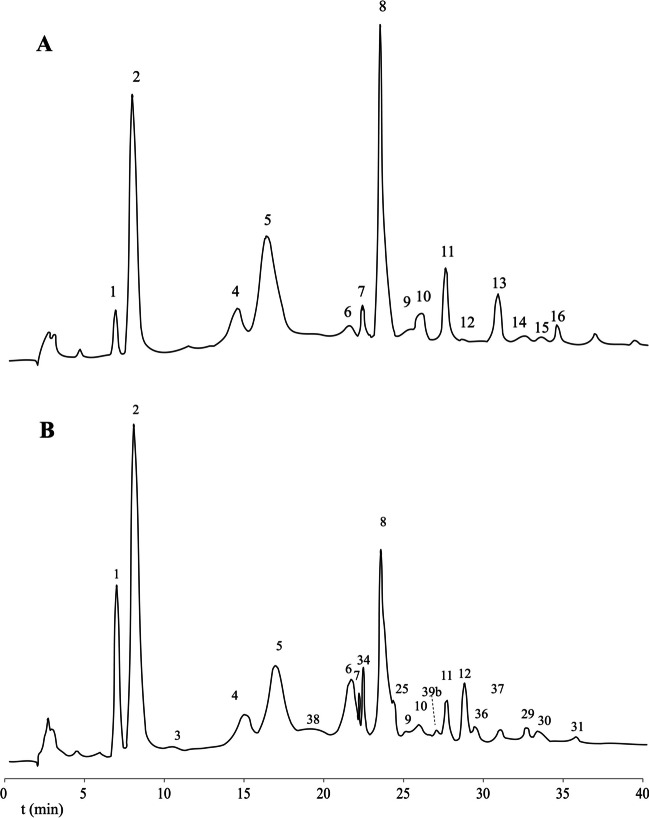
HPAEC-PAD profile of (**A**) Vivinal GOS and (**B**) GOS synthesized by *r*TmBglA from 57%*wt* lactose at 75 °C, using 3.75 U/g lactose for 24 h. Annotated peaks were identified based on references from previous work (Fig. 5) and NMR spectroscopy (Table 3; Fig. 4)

HPAEC-PAD (Fig. 1) profiling of the incubation with lactose showed Glc and Gal release plus a broad range of lactose-derived oligosaccharides. Using the commercial Megazyme TmBglA preparation, several variations in conditions were explored: pH (5 and 8.5), temperature (60 and 90 °C), substrate concentrations (45 and 75%*wt*), and enzyme amounts (2.50 and 5.00 β-galactosidase U/g lactose). As a comparison, also conditions more similar to those used for GOS synthesis with the *Bacillus circulans* β-galactosidase BgaD were used, i.e., 57%*wt* lactose, 75 °C, pH 6.8 with 3.75 β-galactosidase U/g lactose (Yin et al. 2017a). It should be noted that for *B. circulans*, BgaD reactions were performed at 65 °C, due to loss of activity at higher temperatures. HPAEC-PAD profiles (not shown) showed different levels of lactose utilized and different levels of free Gal produced, which indicated differences in *trans*-glycosylation/hydrolysis ratios. These initial chromatograms were used to determine optimal conditions. Visual observations (Supplementary Fig. S2) showed strong browning of the reaction mixtures incubated at 90 °C. Reaction mixtures with 75%*wt* lactose turned darker than reactions using 45%*wt* lactose under the same conditions.

**Fig. 2:**
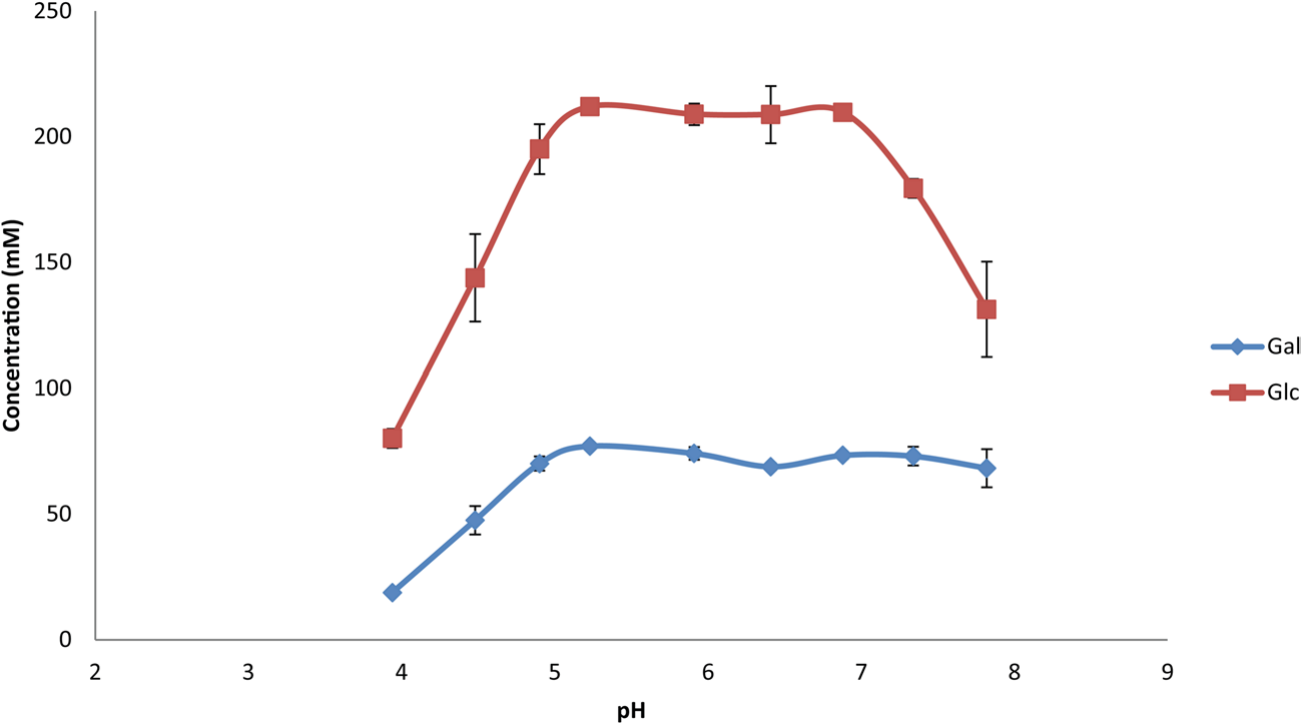
Release of Glc and Gal determined by quantitative HPAEC-PAD profiling in 24-h incubations using 3.75 U/g lactose *r*TmBglA incubated with 57%_wt_ lactose at 75 °C

Further optimization was performed using the recombinant *r*TmBglA at 75 °C. Using HPAEC-PAD quantitation against standard curves of Gal, Glc, lactose, and cellobiose (not shown), yields of GOS and GlcOS could be determined. The highest total GOS production (~ 432 g/L) was obtained with 65%*wt* lactose and highest relative GOS yields (~ 0.68 g GOS/g lactose) at 57%*wt* lactose. Using 57%*wt* lactose at 75 °C, the enzyme showed a broad pH optimum between pH 5.2 and pH 7.0 (Fig. 2). Maximum yield experiments were performed at pH 6.8, using 57%*wt* lactose incubated at 75 °C with 3.75 U/g lactose (72.1%*wt* yield) and 10.0 U/g lactose (63.3%*wt* yield) enzyme activity added (Table 1). Incubation of *r*TmBglA with cellobiose (22 g/L) at 75 °C using the 3.75 U/g cellobiose resulted in a maximum yield of 57.7%*wt* GlcOS (Table 1).

**Table 1:** Yields (%*wt* initial substrate and g/L) as well as yield rate (g/L/h) and yield enzyme efficiency (g/mg BglA and g/mg BglA/h) of GOS synthesis from lactose (57%*wt*), using *r*TmBglA at 3.75 and 10 U/g lactose and GlcOS synthesis from cellobiose (20%*wt*) using *r*TmBglA at 3.75 β-galactosidase U/g cellobiose

	Time (h)	Yield (%*wt*)	Yield (g/L)	Yield (g/L/h)	Yield (g/mg BglA)	Yield (g/mg BglA/h)
GOS (3.75 U/g)	24	72.1	411	17.1	4242	177
GOS (10 U/g)	6	63.3	361	60.1	1396	233
GlcOS (3.75 U/g)	22	57.7	115	5.2	3394	154

pH 6.8, 75 °C

### Enzyme kinetics

The maximum enzyme-specific activity (V*max*) and the Michaelis–Menten constant (*Km*) of *r*TmBglA were determined for lactose and cellobiose using the GOPOD assay for measuring Glc release after 5-min reaction at different substrate concentrations, using Lineweaver–Burk plots (Table 2). The kinetics of the enzyme with lactose showed a biphasic Lineweaver–Burk plot with two separate sets of catalytic parameters (Supplementary Fig. S3). With cellobiose as substrate, the V*max* was 63.1 µmol/min/mg enzyme and *Km* 22.3 mM. With lactose at low concentration, V*max* was 1.6 µmol/min/mg while at high-substrate concentrations, 22.1 µmol/min/mg was observed. The *Km* values with lactose at low- and high-substrate concentrations were 0.4 and 12.9 mM, respectively.

**Table 2:** Enzyme kinetic parameters of *r*TmBglA with different substrates, based on Glc release during the first 5 min of the reaction, monitored by GOPOD analysis

	V*max* (μmol/min/mg)	*Km* (mM)	*kcat* (s^−1^)	*kcat*/*Km* (s^−1^ mM^−1^)
Lactose (low) (*n* = 3)	1.6	0.4	1.4	3.8
Lactose (high) (*n* = 3)	22.1	12.9	19.1	1.5
Cellobiose (*n* = 6)	64.1	22.3	55.6	2.5

pH 6.8, 75 °C

### GOS characterization

**Fig. 3:**
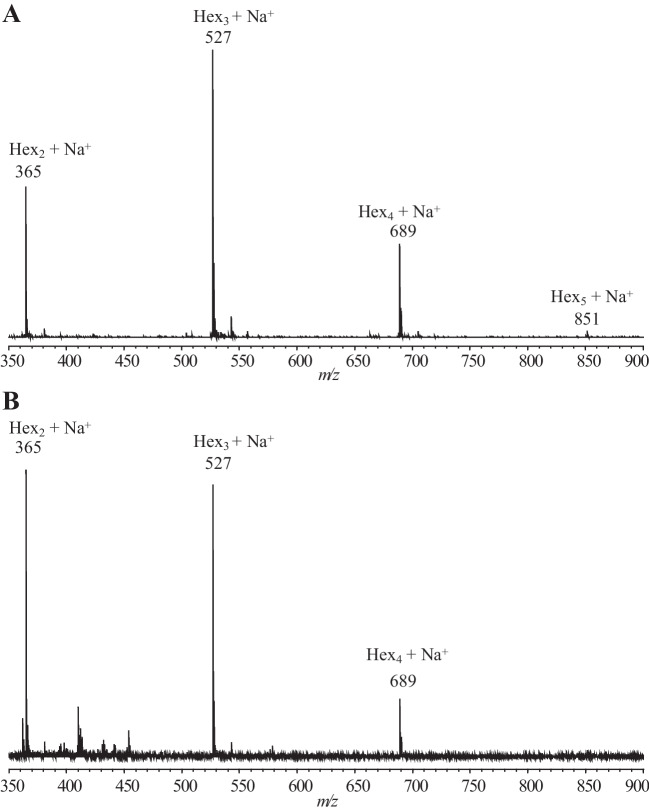
MALDI-TOF–MS spectra of oligosaccharides synthesized by incubation of *r*TmBglA with (**A**) lactose and (**B**) cellobiose. Oligosaccharide peaks are labeled with mass and hexose composition

**Fig. 4:**
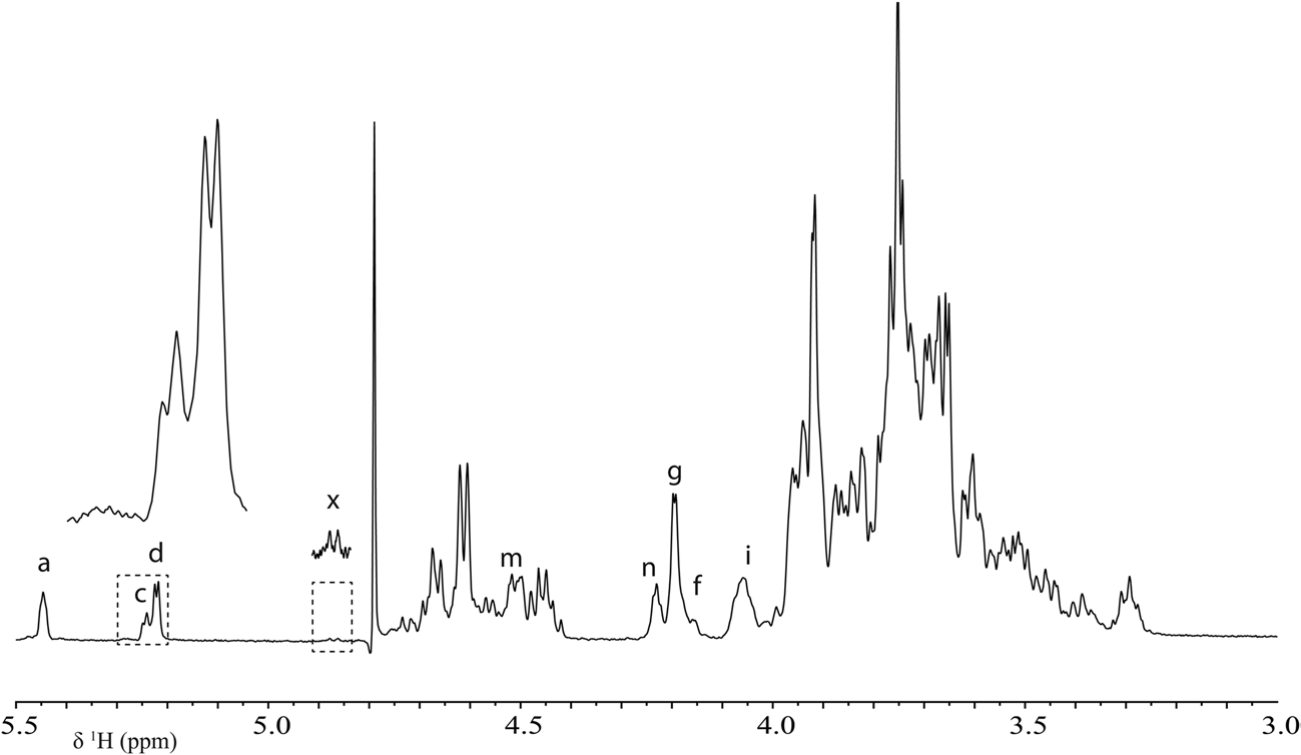
One-dimensional 500 MHz.^1^H NMR spectrum of GOS synthesized by *r*TmBglA from 57%*wt* lactose at 75 °C, using 3.75 U/g lactose for 24 h. Structural-reporter-group signals are annotated (Table 3; Fig. 5) and were used in identification of HPAEC-PAD peaks (Fig. 1)

**Fig. 5:**
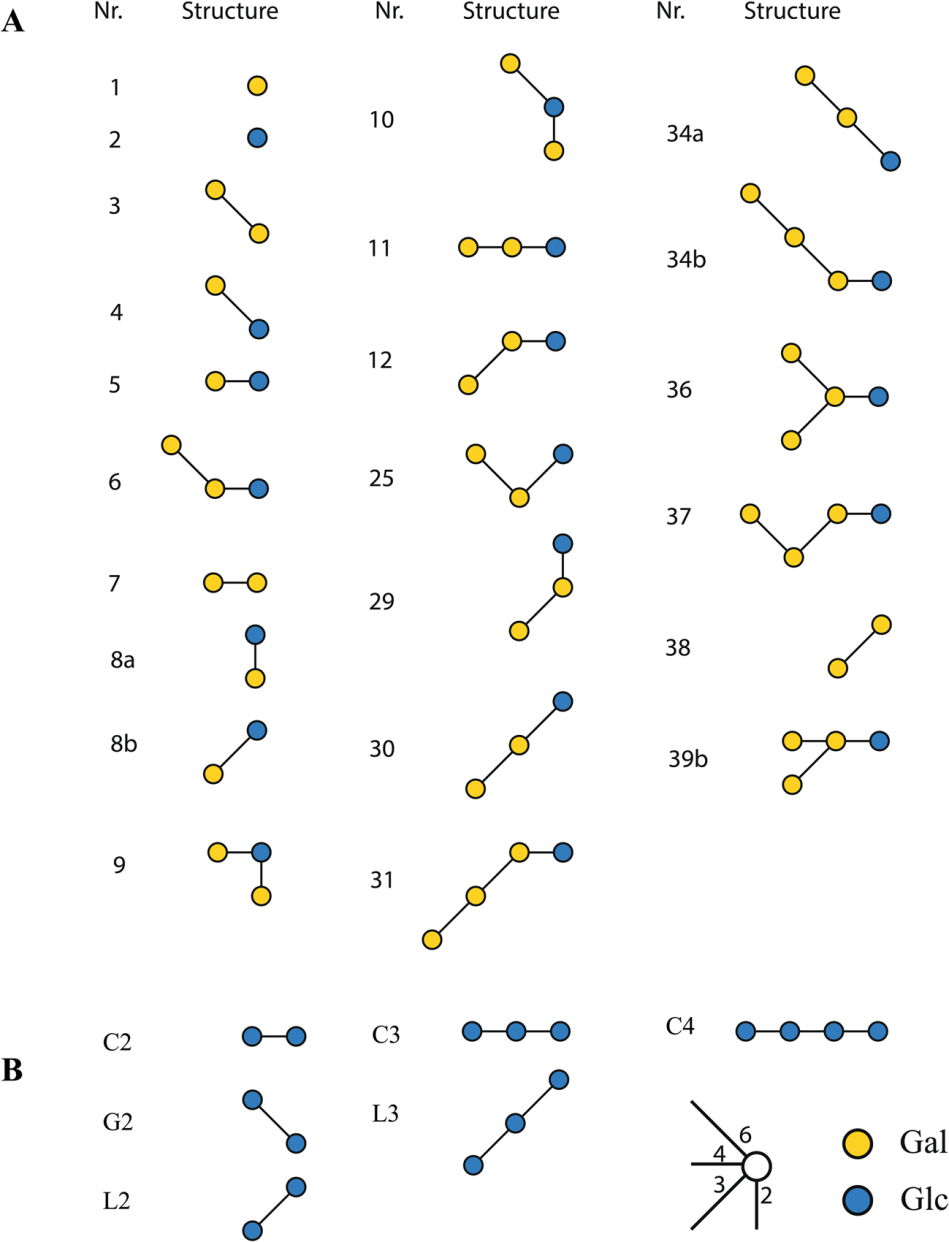
Graphical representation of structures identified in **A** GOS and **B** GlcOS; synthesized by *r*TmBglA from lactose and cellobiose, respectively. Structure numbers match with peaks in Figs. 1 and 6. The structure numbers of GOS used in this figure are the same as in previous publications (van Leeuwen et al. 2016; Yin et al. 2017b, 2018; Kittibunchakul et al. 2020)

MALDI-TOF–MS analysis of the products synthesized from lactose (Fig. 3A) showed peaks at 365, 527, 689, and 851 m*/z*, fitting sodium adducts of di-, tri-, tetra-, and penta-hexose structures, respectively. The HPAEC-PAD profile of maximum yield GOS mixture (Fig. 1) showed a complex peak pattern that was annotated based on retention times in comparison with previously studied GOS mixtures (van Leeuwen et al. 2016; Yin et al. 2017a), combined with NMR spectroscopy data (Fig. 4). NMR structural-reporter-group signals showed specific peaks, supporting the structures identified from the HPAEC-PAD elution times (Table 3; Fig. 5). The main non-lactose DP2 peak corresponded with β-d-Gal*p*-(1 → 3)-d-Glc and β-d-Gal*p*-(1 → 2)- d-Glc. The trisaccharide peak that was most prominent corresponded to β-d-Gal*p*-(1 → 6)-β-d-Gal*p*-(1 → 4)-d-Glc (6′Gallac) and the second peak with β-d-Gal*p*-(1 → 3)-β-d-Gal*p*-(1 → 4)-d-Glc (3′Gallac). There was also evidence for β(1 → 6)-Gal elongation of allolactose, as well as *β*(1 → 6)- and *β*(1 → 3)-Gal elongations of the major disaccharide structures. The lower intensity DP4 fraction showed mainly structures with *β*(1 → 3)- or *β*(1 → 6)-Gal elongations of lactose or combinations of both types of linkages.

**Table 3:** NMR structural-reporter-group signals

Signal code	Chemical shift (ppm)	Explanation
a	5.45	H-1 of a 2-substituted α-d-Glc*p* unit β-d-Gal*p*-(1–2)-α-d-Glc*p*
c	5.23–5.25	H-1 of a 3-substituted α-d-Glc*p* unit[β-d-Gal*p*-(1–3)-α-d-Glc*p*
d	5.22–5.23	H-1 of a 4-, 6-, and/or 1-substituted α-d-Glc*p* unit β-d-Gal*p*-(1–4/6)-α-d-Glc*p*; α-d-Glc*p*-(1–1)-β-d-Gal*p*
f	4.26–4.27	H-6a of a 6-substituted α-d-Glc*p* unit β-d-Gal*p*-(1–6)-α-d-Glc*p*
g	4.27–4.21	H-4 of a 3- and/or 4-substituted β-d-Gal*p* unit β-d-Gal*p*-(1–3/4)-β-d-Gal*p*[-(1-*x*)-]
i	4.05–4.08	H-6a of a 6-substituted β-d-Gal*p* unit β-d-Gal*p*-(1–6)-β-d-Gal*p[*-(1-*x*)-]
m	4.51–4.52	H-1 of a 3-substituted β-d-Gal*p*-(1–4)- unit in a β-d-Gal*p*-(1–3)-β-d-Gal*p*-(1–4)-d-Glc*p* sequence
n	4.23	H-4 of a 3,6-disubstituted β-d-Gal*p*-(1–4)- unit or H-4 of a 3-substituted β-d-Gal*p* unit in a β-d-Gal*p*-(1–6)-β-d-Gal*p*-(1–3)-β-d-Gal*p*-(1–4)-sequence
x	3.83–4.87	H-1 of a 3,4-disubstituted β-d-Gal*p* unit β-d-Gal*p*-(1–3,4)-β-d-Gal*p*-(1-*x*)-

### GlcOS characterization

**Fig. 6:**
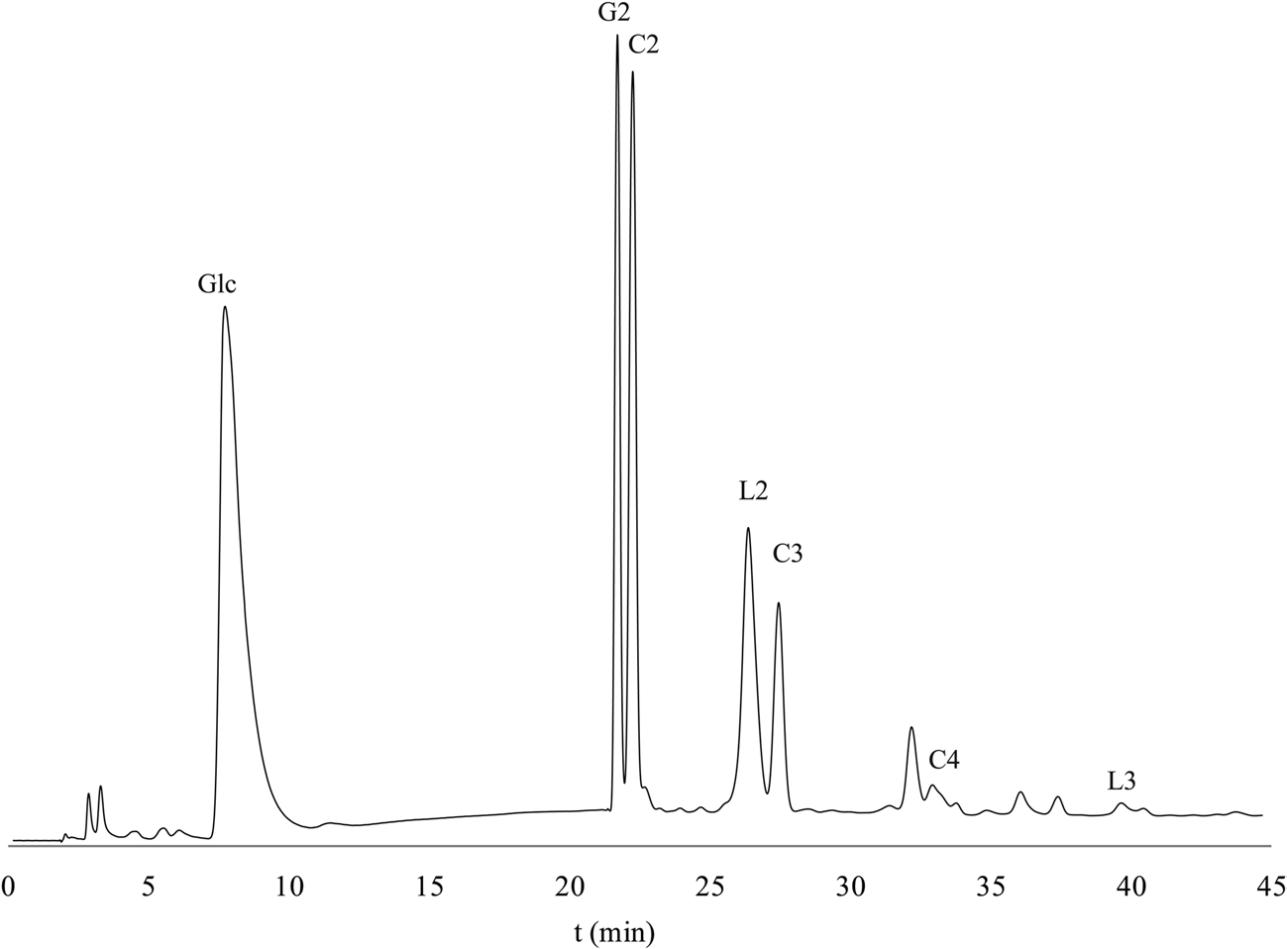
HPAEC-PAD profile of GlcOS synthesized by *r*TmBglA from 20%*wt* cellobiose (C2). Structures are identified based on reference standards, C3: cellotriose [*β*-(1 → 4)], C4: cellotetraose [*β*-(1 → 4)], G2: gentiobiose [*β*-(1 → 6)], L2: laminaribiose [*β*-(1 → 3)] and L3: laminaritriose [*β*-(1 → 3)], matching with identified structures shown in Fig. 5

When incubated with cellobiose, the *r*TmBglA enzyme synthesized various GlcOS structures. The MALDI-TOF–MS spectrum (Fig. 3B) showed peaks at 365, 527, and 689 m*/z* indicating sodium-adduct peaks of Hex_2_, Hex_3_, and Hex_4_ structures, respectively. Besides glucose and cellobiose, three major peaks and several minor peaks were observed in the HPAEC-PAD profile (Fig. 6).

The major structures formed are β-d-Glc*p*-(1 → 6)-d-Glc (gentiobiose; G2), β-d-Glc*p*-(1 → 3)-d-Glc (laminaribiose; L2), and β-d-Glc*p*-(1 → 4)-β-d-Glc*p*-(1 → 4)-d-Glc (cellotriose; C3). Minor amounts of laminaritriose (L3) and cellotetraose (C4) were also observed. A medium-sized peak was found at retention time 32.6 min between the elution times for C3 and C4. Gentiotriose (G3) and gentiotetraose (G4) standards were not available, but are expected to elute slightly before C3 and C4, respectively, based on the relative elution positions of G2 and C2. Possibly, G3 elutes at the same retention time as L2, for which a standard was available, and the peak at 32.6 min might be G4. Comparing the relative intensities of the G2 and C2 peaks, and the C3 and C4 peaks, it is also possible that the peak at 32.6 min is a *β*(1 → 3) elongation of C2 or a *β*(1 → 6) elongation of L2 or C3. Other minor peaks were not identified, from MALDI-TOF–MS analysis; however, only di- up to tetra-hexose structures were observed. The elution positions of these peaks lie in between cello-oligosaccharide and laminari-oligosaccharide standards, indicating tri- and tetrasaccharides with combinations of *β*(1 → 3), *β*(1 → 4), and *β*(1 → 6)-linkages. These cellobiose-derived *trans*-glucosylation products show a similar linkage preference for *β*(1 → 3) and *β*(1 → 6) linkages, as was observed for *trans*-galactosylation with lactose.

## Discussion

The incubation with the commercial TmBglA preparation showed *trans*-glycosylation activity (Fig. 1). Initial optimization experiments showed that despite high thermostability, there is a limit to the gain of higher enzyme activity. When incubated at 90 °C, the enzyme showed clear activity, but the product showed significant browning, most likely a result of Maillard reaction. A higher dose of enzyme also increased browning, fitting the Maillard hypothesis. Incubations with 75%*wt* lactose at 60 °C showed difficulty in keeping lactose dissolved, as evidenced by large amounts of precipitate in the product (Supplementary Fig. S2). After 24-h incubation, the slurry still produced GOS; higher doses of enzyme resulted in less precipitated material in the final product. HPAEC-PAD analysis (not shown) of all the investigated incubation conditions clearly showed reduced lactose consumption ratios when 75%*wt* lactose was incubated at 60 °C. This indicates that a slurry or syrup can still be used efficiently in GOS synthesis, but high amounts of precipitated lactose limit the reaction.

The SDS-PAGE analysis of the commercial TmBglA preparation revealed multiple bands, indicating impurities. Since it cannot be excluded that an impurity is responsible for the activity towards lactose, the recombinant gene was ordered in a plasmid (GeneArt) and used to express *r*TmBglA, which was successfully purified (Supplementary Fig. S1). The recombinant enzyme appeared with a higher MW ~ 56 kDa on the SDS-PAGE gel, which is most likely the effect of the His6-tag, which has been described to disproportionately affect mobility in the gel (Niu and Guiltinan 1994). Comparison of GOS synthesized from lactose with the Megazyme TmBglA preparation and the purified *r*TmBglA enzyme showed the same product profiles (Supplementary Fig. S4). The *trans*-glycosylase activity with lactose thus is not based on a contaminant protein in the Megazyme TmBglA preparation, but is an inherent activity of the TmBglA enzyme itself. This observation also fits previous reports showing activity of this TmBglA towards lactose (Gabelsberger et al. 1993) and *trans*-glycosylase activity of a similar enzyme from *T. naphthophila* (Yang et al. 2018). The latter reports only production of 3′-galactosyllactose as a single product. Neither study has evaluated oligosaccharide synthesis with cellobiose as substrate.

### Enzyme activity and kinetics

The kinetics of the enzyme with lactose showed a biphasic Lineweaver–Burk (Supplementary Fig. S3; Table 2). One set of kinetic parameters is for low-substrate concentrations, and one set is for high-substrate concentrations. Such biphasic plots also were observed for other GOS-synthesizing enzymes, e.g., for the β-galactosidase from *B. circulans* BgaD (Song et al. 2011b, 2011a; Bultema et al. 2014). Possibly, at higher substrate concentrations, the products of the *trans*-glycosylation reaction significantly influence the rate of Glc release from lactose. For cellobiose, such biphasic kinetics was not observed. This may indicate a more limited *trans*-glycosylation activity towards cellobiose. It is apparent that the catalytic rate with cellobiose, based on release of Glc, is significantly higher than with lactose. One early study showed that the specific activity with cellobiose is higher than with lactose (Gabelsberger et al. 1993). Gabelsberger et al. compared the reactions with cellobiose and lactose, showing 42 and 28%, respectively, of the activity with pNP-β-d-Glc (100%) (Gabelsberger et al. 1993). Gabelsberger et al. determined relative activities using 40 mM lactose and cellobiose at pH 6.2 and 75 °C. At low-substrate concentrations, the catalytic efficiency *kcat*/*Km* of *r*TmBglA is higher for lactose than for cellobiose, while at higher concentrations of lactose, the enzyme is less efficient than at low lactose concentrations. Further reactions of the enzyme with *trans*-glycosylation products as donor already in the early reaction phase do not result in release of Glc and were therefore not detected in both the reaction with lactose as well as with cellobiose. This may result in an underestimated catalytic rate and thereby an underestimated catalytic efficiency of the enzyme. It should be noted that the *Km *values with both cellobiose, as well as lactose, are relatively high, indicating low-substrate affinity. Possibly, this results in lower activity towards the remaining substrate near the end of the reaction when the substrates near depletion.

Yields were determined by quantitative HPAEC-PAD analysis of Glc and Gal, as well as residual lactose (Table 1; chromatograms not shown). Using *r*TmBglA enzyme, we obtained a maximal yield of 72.1%*wt* GOS from 570 g/L lactose, with only 10.5%*wt* remaining lactose. To the best of our knowledge, this is the highest yield obtained so far in enzymatic GOS production. The GOS yield observed for the commercially used *B. circulans* BgaD is 63.3%*wt* GOS with 21.2%*wt* remaining lactose after 22 h (Benjamins et al. 2014). Between 24 and 48 h, the GOS yield declined again, probably due to hydrolytic activity of the enzyme towards the GOS products. When the enzyme concentration was increased to 10 U/g lactose, a maximum GOS yield of 63.3%*wt* was obtained in 6-h reaction time (Table 1). The reduced yield at higher enzyme concentration is likely the effect of limited lactose availability per enzyme molecule, resulting in a relatively higher hydrolysis ratio. Previous work using immobilized *T. maritima* BglA showed a GOS yield of ~ 24%*wt* with free enzyme and 28%*wt* with immobilized enzyme (Alnadari et al. 2021). The latter study, however, used 200 g/L lactose and a relatively high enzyme load (270 U/mL) and screened for products by TLC analysis. These conditions are likely more conducive to hydrolysis than *trans*-glycosylation, which negatively influences GOS yields.

The yield efficiency of GOS in g/L is higher for the incubation with *r*TmBglA β-galactosidase activity at 3.75 U/g lactose, while the GOS yield in g/L/h is 3.5 times higher with *r*TmBglA at 10 U/g lactose (Table 1). From the V*max* with lactose (Table 2), the yield in g GOS per g enzyme can be calculated, showing that the incubation with 3.75 U/g lactose is much more efficient in terms of enzyme use. When the cost of the enzyme is the most important economic cost factor, GOS synthesis at 3.75 U/g lactose is most favorable. However, if bioreactor availability or production times are a limiting factor, it may be more economical to shorten reaction times with higher enzyme doses to increase the total product versus time ratio. Using similar enzyme-to-substrate conditions, from cellobiose, a 57.7%*wt* maximum yield was achieved. This is a promising relative yield, but relatively low on absolute conversion with 115 g/L and only 5.2 g/L/h conversion. Enzyme efficiency was calculated from the total yields and amount of enzyme added. In terms of enzyme efficiency, a yield of 3394 g GlcOS/mg enzyme was still ~ 80% compared with GOS yields at 4242 g GOS/mg enzyme (Table 1).

The *r*TmBglA GOS product mixture showed 20 GOS structures with a mixture of *β*(1 → 3), *β*(1 → 4), and *β*(1 → 6)-linkages, as well as remaining lactose, and free Gal and Glc. Previous studies have shown commercial GOS products with a focus towards *β*(1 → 3) and *β*(1 → 6), towards *β*(1 → 4) and *β*(1 → 6), or towards a single linkage preference. In case of commercial GOS preparations, some are the result of a combination of two enzymes. Previously, the β-galactosidase BgaD from *B. circulans* was adapted by a single-point mutation to produce GOS with a combined *β*(1 → 3) and *β*(1 → 4) linkage preference (Yin et al. 2017b). That study was the first example of a product with that combination of structures, culminating in structure **39b**, which was observed there for the first time (Yin et al. 2017b). Our results show that *r*TmBglA is a naturally occurring enzyme capable of synthesizing *β*(1 → 3) and *β*(1 → 6) linked GOS from lactose, with also some *β*(1 → 4) epitopes. This combination of three types of GOS elongation, *β*(1 → 3), *β*(1 → 4), and *β*(1 → 6), has not been observed previously, rendering *r*TmBglA a very interesting example of a GOS-synthesizing enzyme.

When incubated with cellobiose, the product spectrum is less complex, showing mainly di- and trisaccharides. The maximum concentration of cellobiose that could be included in a solution or slurry (~ 20%*wt*) is significantly lower than for lactose (~ 65%*wt*) at 75 °C, limiting the cellobiose availability. Despite this limitation, a good conversion to cellobiose-derived di-, tri-, and tetrasaccharides was achieved, with mainly *β*(1 → 3) and *β*(1 → 6) elongations, similar to the GOS product with lactose as substrate. The relatively high concentrations of DP2 products gentiobiose and laminaribiose suggest that initially released free Glc is a good acceptor molecule. Just as with lactose as substrate, the cellobiose incubation also shows some *β*(1 → 4) elongations, such as the formation of cellotriose and cellotetraose.

This paper shows that the β-glucosidase BglA from *T. maritima* is a versatile, thermostable enzyme capable of synthesizing a variety of different oligosaccharide mixtures at higher yields than observed so far. Based on previous literature, the GOS mixture from lactose, containing mainly *β*(1 → 3) and *β*(1 → 6)-linked Gal residues, are very promising bifidogenic products.

## Supplementary Information

Below is the link to the electronic supplementary material.

Supplementary file1 (PDF 554 KB)

## Data Availability

Data will be made available on reasonable request.
