# Supplementary material for: Kinetics and products of Thermotoga maritima β-glucosidase with lactose and cellobiose
Source: Appl Microbiol Biotechnol. 2024 May 29;108(1):349. doi: 10.1007/s00253-024-13183-6 (PMC11136819; doi:10.1007/s00253-024-13183-6)
Supplement: Supplementary file 1 — Supplementary file1 (PDF 554 KB) [file 253_2024_13183_MOESM1_ESM.pdf]

Supplementary materials belonging to Applied Microbiology and Biotechnology article:

Kinetics and products of *Thermotoga maritima* β-glucosidase with lactose and cellobiose

Geert A. ten Kate[1,a], Peter Sanders[2], Lubbert Dijkhuizen[1,b], Sander S. van Leeuwen[1,3,*]

[1] Microbial Physiology, Groningen Biomolecular Sciences and Biotechnology Institute (GBB), University of Groningen, Nijenborgh 7, 9747 AG, Groningen, The Netherlands

[2] Eurofins Expertise Centre for Complex Carbohydrates and Chemistry, PO Box 766, 8440 AT, Heerenveen, The Netherlands

[3] Department of Laboratory Medicine, University of Groningen, University Medical Center Groningen, Hanzeplein 1, EA30, 9713 GZ, Groningen, The Netherlands

[a] Current Address : Royal FrieslandCampina, Stationsplein 4, 3818 LE, Amersfoort, The Netherlands

[b] Current Address : CarbExplore Research BV, Zernikelaan 8, 9747 AA, Groningen, The Netherlands

* Corresponding author : s.s.van.leeuwen@rug.nl

Sequence information

Sequence GeneArt *E. coli* optimized rTmBglA *E. coli* with His6-tag

1 ATGCATCATC ACCATCATCA TGGTGGTGGT ATGAACGTTA AAAAGTTTCC GGAAGGTTTT CTGTGGGGTG TTGCAACCGC

81 AAGCTATCAG ATTGAAGGTA GTCCGCTGGC AGATGGTGCA GGTATGAGCA TTTGGCATAC CTTTAGCCAT ACACCGGGTA

161 ATGTTAAAAA TGGTGATACC GGTGATGTTG CCTGCGATCA TTATAATCGT TGGAAAGAAG ATATCGAGA TCATCGAAAA

241 ACTGGGCGTT AAAGCATATC GTTTTAGCAT TAGCTGGCCT CGTATTCTGC CGGAAGGCAC CGGTCGTGTT AATCAGAAAG

321 GTCTGGATTT CTATAACCGC ATTATTGATA CCCTGCTGGA AAAAGGTATT ACCCCGTTTG TTACCATCTA TCATTGGGAT

401 CTGCCGTTTG CACTGCAGCT GAAAGGTGGT TGGGCAAATC GTGAAATTGC AGATTGGTTT GCAGAATATA GCCGTGTGCT

481 GTTTGAAAAT TTTGGTGATC GTGTCAAAAA CTGGATCACC CTGAATGAAC CGTGGGTTGT TGCCATTGTT GGTCATCTGT

561 ATGGTGTTCA TGCACCGGGT ATGCGTGATA TTTATGTTGC ATTTCGTGCC GTTCATAATC TGCTGCGTGC ACATGCACGT

641 GCCGTTAAAG TTTTTCGTGA AACCGTTAAA GATGGCAAAA TTGGCATCGT GTTTAACAAC GGTTATTTTG AACCGGCAAG

721 CGAGAAAGAA GAGGATATTC GCGCAGTTCG TTTTATGCAC CAGTTTAATA ACTATCCGCT GTTTCTGAAT CCGATCTATC

801 GTGGTGATTA TCCGGAACTG GTTCTGGAAT TTGCACGTGA ATATCTGCCC GAGAACTATA AAGATGATAT GAGCGAAATC

881 CAAGAGAAAA TCGACTTTGT GGGCCTGAAC TATTATAGCG GTCATCTGGT TAAATTTGAT CCGGATGCAC CGGCAAAAGT

961 TAGCTTTGTT GAACGTGATC TGCCGAAAAC CGCAATGGGT TGGGAAATTG TTCCTGAAGG TATTTATTGG ATCCTGAAAA

1041 AGGTGAAAGA GGAATATAAT CCGCCTGAGG TGTATATTAC CGAAAATGGT GCAGCATTTG ATGATGTTGT TAGCGAAGAT

1121 GGTCGTGTGC ATGATCAGAA TCGTATCGAT TATCTGAAAG CCCATATTGG TCAGGCATGG AAAGCAATTC AAGAAGGTGT

1201 TCCGCTGAAA GGCTATTTTG TTTGGAGCCT GCTGGATAAT TTTGAATGGG CAGAAGGTTA TAGCAAACGC TTTGGTATTG

1281 TGTATGTGGA TTACAGCACC CAGAAACGCA TTGTTAAAGA TAGCGGTTAT TGGTATAGCA ACGTGGTGAA AAATAACGGC

1361 CTGGAAGATT AG

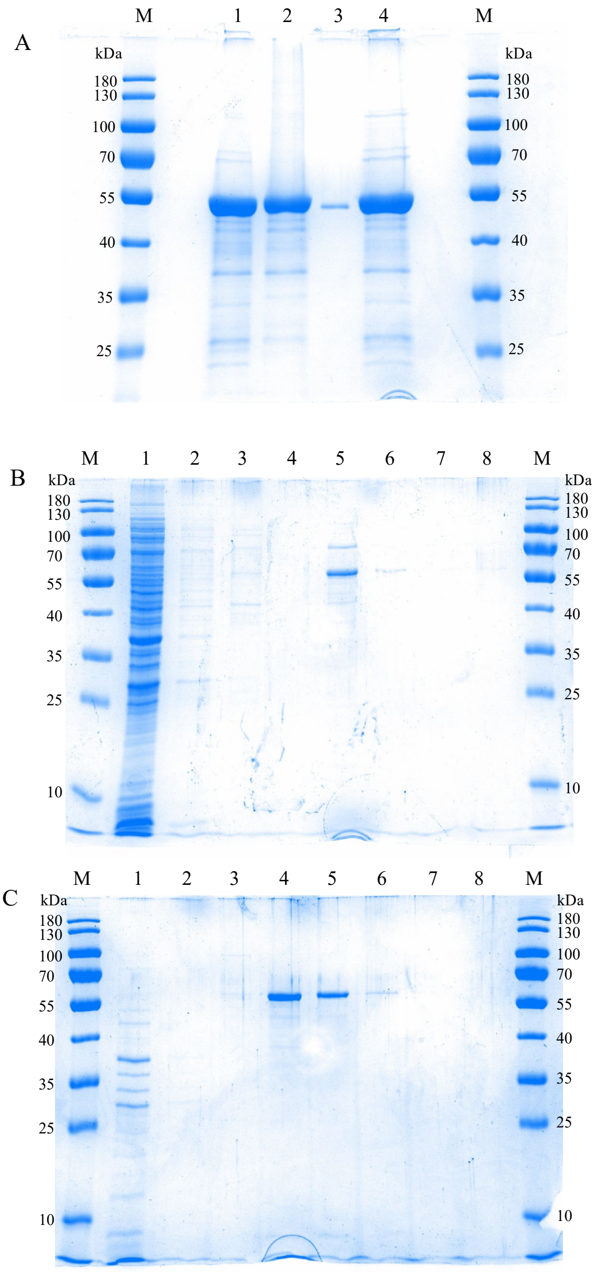

Fig. S1. SDS-PAGE analysis of TmBglA purification: A) Gel of Megazyme TmBglA preparation lanes: M-marker, 1. Undiluted, 2. 10x diluted, 3. 100x diluted, 4. undiluted , B) Gel of *r*TmBglA after Ni-NTA purification; Lanes: M. marker, 1-3 Ni-NTA wash fractions, 4-8 Ni-NTA elution fractions and C) Gel of Ni-NTA fractions after precipitation of heat-denatured proteins; Lanes: M. marker, 1-3 Ni-NTA wash fractions, 4-8 Ni-NTA elution fractions.

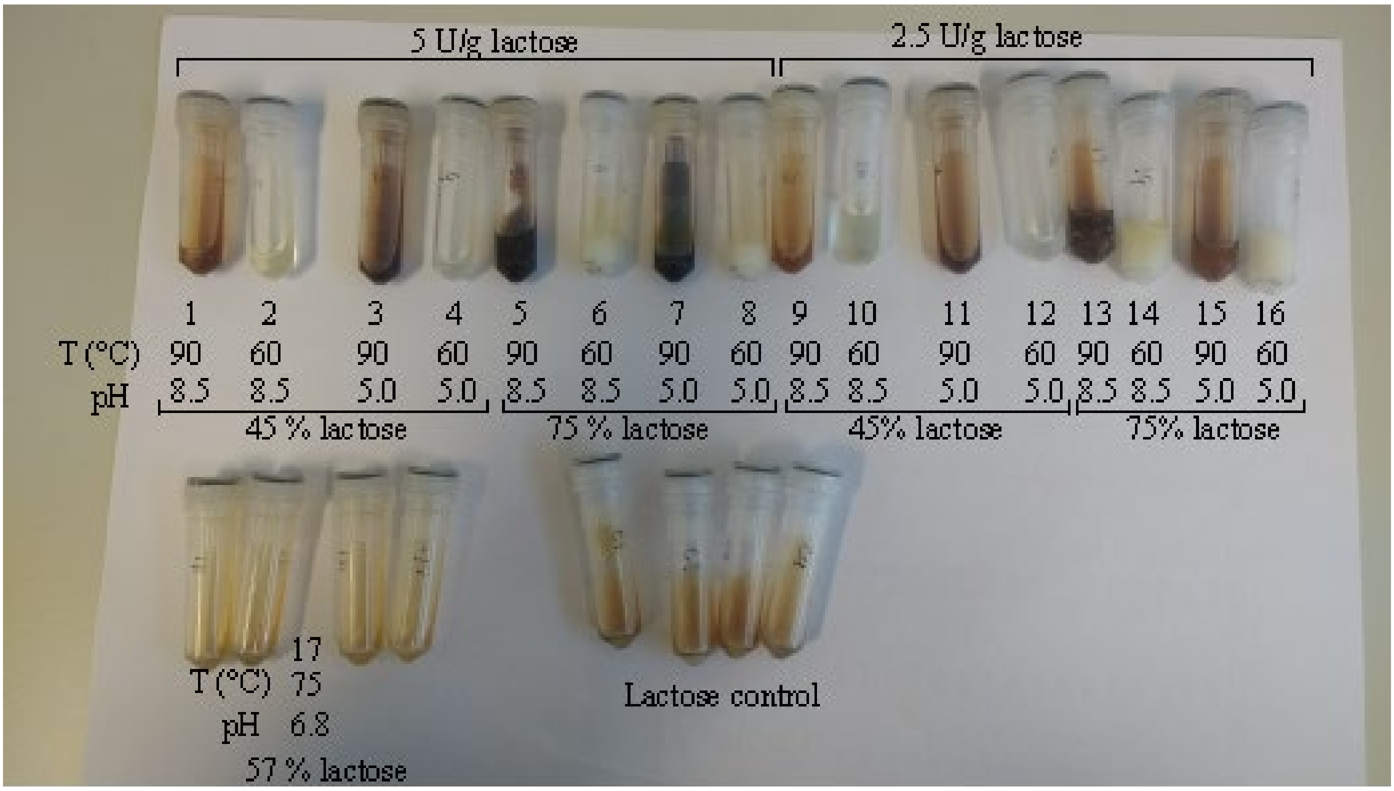

Fig. S2. Photograph of reaction tubes of different incubation conditions of lactose with *r*TmBglA enzyme incubated 24h at different temperatures and pH. Lactose controls are incubated without enzyme.

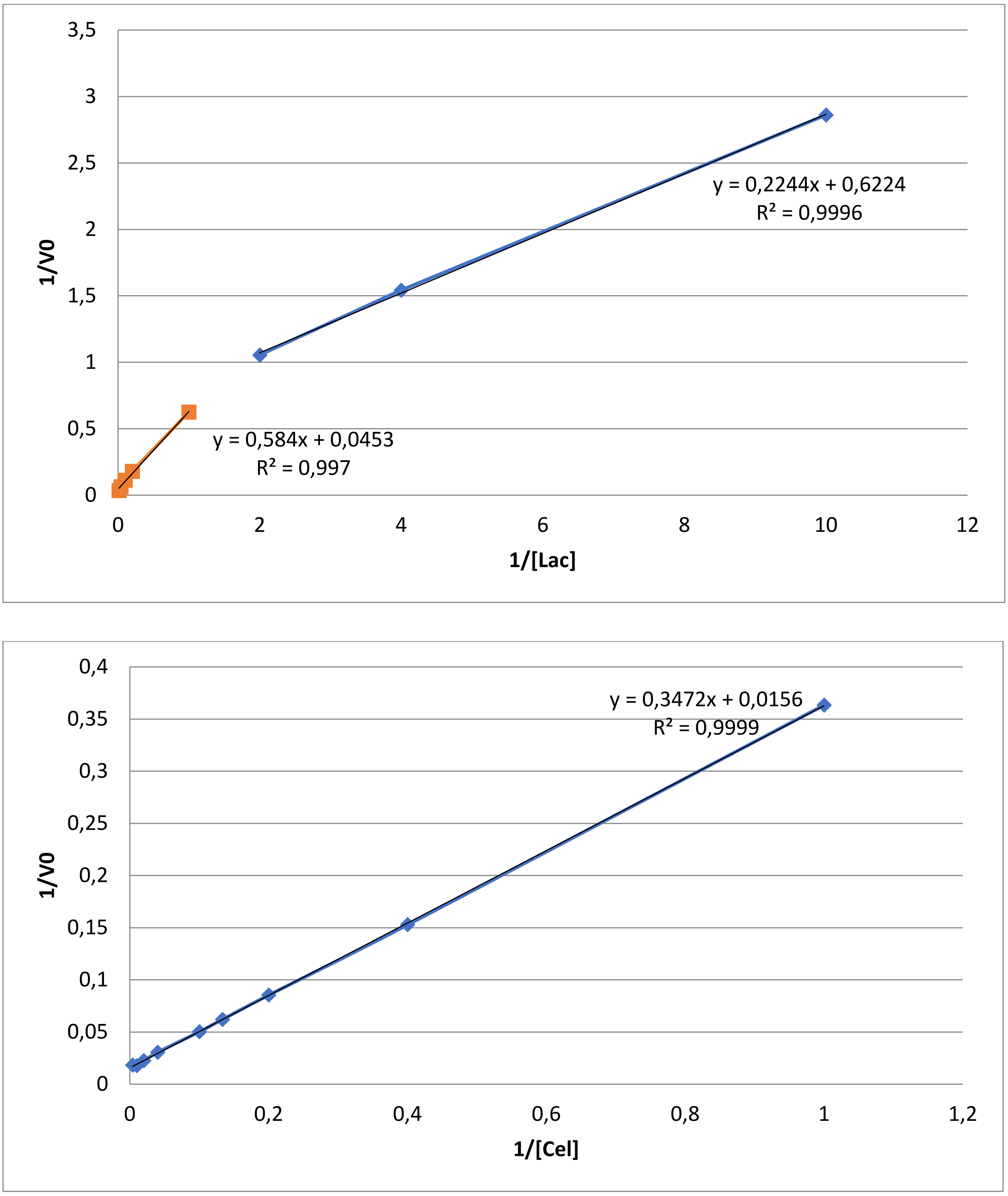

Fig. S3. Lineweaver-Burk plots of 3.38 µg *r*TmBglA incubated in 150 µL with lactose 0.1-75 mM (top) and cellobiose 1-250 mM (bottom).

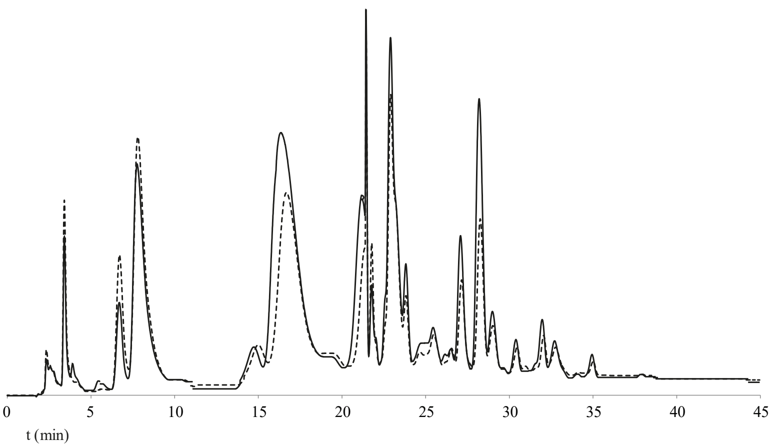

Fig. S4. Comparison of HPAEC-PAD chromatograms of products formed from lactose (57 %*wt*) incubated at 75 °C with TmBglA from Megazyme (dashed line) and the recombinant *r*TmBglA after heat treatment and Ni-NTA purification (solid line).
